# Comparison of Risk Factor between Lacunar Stroke and Large Artery Atherosclerosis Stroke: A Cross-Sectional Study in China

**DOI:** 10.1371/journal.pone.0149605

**Published:** 2016-03-02

**Authors:** Pu Lv, Haiqiang Jin, Yuanyuan Liu, Wei Cui, Qing Peng, Ran Liu, Wei Sun, Chenghe Fan, Yuming Teng, Weiping Sun, Yining Huang

**Affiliations:** Department of Neurology, Peking University First Hospital, Beijing, China; Shanghai Institute of Hypertension, CHINA

## Abstract

**Background:**

Stroke is the second most common cause of mortality in China. Although most subtypes of ischemic stroke share similar risk factors, they have different etiologies. Our study aimed to evaluate the different risk factor profiles between the stroke subtypes, lacunar infarcts (LI) and large-artery atherosclerosis (LAA), and clarify the characteristics of current acute ischemic stroke in China.

**Methods:**

In this cross-sectional study, we analyzed the clinical characteristics of 1982 patients with acute ischemic stroke who were admitted to the neurology department at the Peking University First Hospital between 2007 and 2014. Ischemic stroke was further classified into LAA, LI, cardioembolism (CE) and undetermined causes of infarction (UDI) according to TOAST classification. Demographic characteristics, risk factors, as well as the findings of laboratory and imaging tests of 1773 patients with LAA and LI, were analyzed by univariate and multivariate logistic analysis.

**Results:**

Of the 1982 ischemic stroke patients included in this study, 1207 were diagnosed with LAA, 566 with LI, 173 with cardioembolism (CE) and 36 with undetermined causes of infarction (UDI). By comparing the risk factors in multivariate logistic regression analysis, hypertension [odds ratio (OR) = 1.832] and white matter leukoaraiosis (WML) (OR = 1.865) were found to be more strongly correlated with LI than LAA. Low density lipoprotein- cholesterol (LDL-c) (OR = 0.774) were more strongly related to LAA than LI.

**Conclusions:**

This study found that hypertension and WML were more strongly correlated with LI than LAA. LDL-c was more strongly related to LAA than LI.

## Background

Stroke is the second most common cause of mortality in China[1]. According to a long-term follow-up study, the incidence of stroke increased during the past decades [2]. Because of variances in occurrence, treatment, and prognosis among different stroke subtypes, it is important to explore the risk factor of each subtype and implement preventive measures. Ischemic stroke is the most common stroke subtype in China. Currently, the most frequently used classification of stroke is the Trial of Org 10172 in Acute Stroke Treatment (TOAST) classification, which indicates the risk factors for each stroke subtype definition[3, 4]. Lacunar infarction (LI) refers to a small cortical infarction (in the chronic phase, with a diameter of <15 mm) located in the internal capsule, basal ganglia, corona radiate, thalamus or brainstem, which is caused by the occlusion of a perforating artery[5]. In China, LI and cerebral hemorrhage are far more common than in Western populations[6, 7]. According to a hospital-based study, LI accounted for 38.2% of all cases of cerebral infarction, and cerebral hemorrhage accounted for 31.3% of all stroke cases[8]. Therefore, exploring risk factors for small vessel disease (SVD) is the priority of stroke prevention in China.

A previous study showed that large-artery atherosclerosis (LAA) and SVD have the same risk factors[9]. The most studied risk factors for SVD are age, hypertension and diabetes. Based on pathological studies, larger LIs without white matter leukoaraiosis (WML) involve an atheroma at the origins or proximal portions of the larger (200–800 μm in diameter) perforating arteries[10]. Multiple smaller LIs with WML involve a diffuse arteriopathy of the smaller perforating arteries (40–200 μm in diameter).

The pathogenesis of SVD is still in debate. However, it is thought that if atherosclerosis plays a part in SVD development, LAA and SVD should share similar risk factor profiles. Thus, comparing the risk factor profile of these two subtypes of stroke may contribute to elucidate their pathology. Therefore, our study aimed to evaluate the different risk factor profiles between LI and LAA.

## Methods

In this cross-sectional study, we consecutively recruited 1982 acute ischemic stroke patients within 2 weeks of symptom onset admitted to the neurology department of Peking University First Hospital between 2007 and 2013. We enrolled patients aged> 18 years with clinical symptoms of stroke and a confirmed brain CT-scan or MRI diagnosis of cerebral infarction at hospital discharge according to the criteria of the World Health Organization, National Institute of Neurological Disorders and Stroke, or the definition of stroke from the Atherosclerosis Risk in Communities (ARIC) Study[11–13]. Patients with severe renal disease (Scr>707umol/L) were not included. The following variables were recorded for each patient: age, sex, living habits (smoking and alcohol abuse, defined as alcohol consumption within the 3 months before stroke onset), and medical history (hypertension, diabetes, prior stroke/transient ischemic attack [TIA], prior coronary heart disease [CHD; includes any history of heart attack/myocardial infarction or angina]) and previous therapy (includes anti-hypertensive drug use, anti-diabetic therapy, anti-platelet drug use and statin use). History of hypertension was defined as either a blood pressure reading of >140/90 mm Hg from records of the hospital or from patient recall. History of diabetes was defined either as records of fasting blood glucose > 7.0mmol/L, postprandial blood glucose > 11.1 mmol/L or used to on anti-diabetic treatments.

Onset measurements included systolic blood pressure (SBP), diastolic blood pressure (DBP), biochemical tests (hemoglobin, albumin, serum creatinine, total cholesterol, serum triglycerides, low-density lipoprotein cholesterol (LDL-c), high-density lipoprotein cholesterol(HDL-c), homocysteine), and WML (defined as neuroimaging abnormalities of the white matter in bilaterally patchy or diffuse areas of hypodensity on computed tomography or hyperintensity on T2-weighted or fluid-attenuated inversion recovery magnetic resonance imaging)[14, 15]. Blood pressure (BP) wasmeasured at the time of recruitment. BP was the average of two readings at rest. The systolic blood pressure (SBP) and diastolic blood pressure (DBP) were recorded for each subject. Blood samples were drawn from the peripheral vein in the morning after overnight fast. Tubes were centrifuged at 3000g for 10 min at room temperature. All the blood variables were measured using an autoanalyzer (Hitachi 747; Hitachi, Tokyo, Japan) at the laboratory department of the Peking University first hospital.

Ischemic stroke was further classified into LAA, LI, cardioembolism (CE) and undetermined causes of infarction (UDI) according to TOAST classification. Patients with LI were defined as having lacunar syndrome and signs, and brain neuroimaging evidence of infarct size of ≤1.5 cm at a typical location[5, 16]. Patients with LAA were defined as having clinical symptoms of stroke with brain imaging evidence of infarct size of >1.5 cm, no definite cardioembolic source, but moderate to severe extra or intracerebral arterial stenosis, or infarction of other determined causes. The same criteria were applied for CE in addition to evidence of a possible source of emboli, such as atrial fibrillation, valvular heart disease, or history of acute myocardial infarction. Infarcts that did not meet the above criteria were defined as UDI.

This study was approved by the ethics committee of Peking University First Hospital. All study procedures were performed in accordance with the ethical standards laid down in the 1964 Declaration of Helsinki and its later amendments. We asked each patient whether they wished to be included in our study, and got their permission. We recorded the participants’ responses in a table. The ethics committee approved this consent procedure. Thus, all patients gave their informed consent prior to their inclusion in the study.

### Statistical methods

All the 1982 patients with ischemic stroke were classified according to their stroke subtype: LI, LAA, CE and UDI groups. Distribution of patient characteristics and risk factors among stroke subtypes were compared. Numerical variables were described as mean ± standard deviation (SD) and analyzed by the Student’s t-test or Wilcoxon rank sum test. Categorical variables were described as percentage and analyzed based on the chi-square test or Fisher’s exact test. Demographic characteristics, risk factors, as well as the findings of laboratory and imaging tests of 1773 patients with LAA and LI, were analyzed by univariate logistic regression analysis. A multivariate logistic regression analysis was also performed to identify risk factors between LI and LAA stroke. The variables found to be associated with LI in the univariate analysis by p<0.20 were further tested by binary logistic regression in the multivariate analysis. Varibles were underwent correlation analysis. If the r>0.8 between two variables and the P< 0.05, the two variables were considered highly correlated. One of them was excluded from the logistic model (data in S1 Table). According to this criterion, total cholesterol showed a highly correlation with LDL-c, so it was excluded in the multivariate logistic model for LDL-c and other variables. Besides, in order to explore the impact of total cholesterol on stroke subtype, a model which excluded LDL-c was built for the analysis of total cholesterol while other variables remained unchanged. P<0.05 was considered statistically significant. All statistical analyses were performed using the R software package Version 3.0.2.

## Results

A total of 1982 ischemic stroke patients treated at our institution between 2007 and 2014 were included in the analysis. Of these, 1207 were diagnosed with LAA (60.9%), 566 with LI (28.6%), 173 with CE (8.7%) and 36 (1.8%) with UDI. Patients with ischemic stroke had a mean age of 67 ± 13 (range, 20–96) years. Of the total patients, 1299 were male (65.5%) and 683 were female (34.5%) (Table 1).

**Table 1:** Characteristics of patients stratified by ischemic stroke (IS) subtype.

		Etiologic subtypes of IS			
	All IS	LI	LAA	CE	UDI
N	1982	566(28.6%)	1207(60.9%)	173(8.7%)	36(1.8%)
Male, N	1299(65.5%)	375(66.3%)	824(68.3%)	75(43.4%)	25(69.4%)
Female, N	683(34.5%)	191(33.7%)	383(31.7%)	98(56.6%)	11(30.6%)
Age, years	67±13	69±12	66±13	70±10	46±7

Values are reported as mean±SD or n (%) of subjects.

IS: ischemic stroke; LI: lacunar infarct; LAA: large artery atherosclerosis; CE: cardioembolism; UDI: undetermined causes of infarction.

Table 2 shows the prevalence of risk factors between patients with LI and LAA subtypes. Regarding cerebral infarction subtype, age, history of coronary heart disease (CHD), history of hypertension, history of diabetes, anti-hypertensive drug use, anti-diabetic therapy, anti-platelet drug use, WML, SBP, DBP, hemoglobin, serum total cholesterol, and LDL-c were significantly different between patients with LI and LAA. Patients with LAA were younger on average than those with LI (66 vs. 69, P<0.001). The LI group had elevated proportions of CHD history (26.5% vs. 21.8%, P = 0.033), hypertension history (69.8% vs. 53.3%,P<0.001), diabetes (33.7% vs. 23.4%, P<0.001) compared with the LAA group, and these difference between groups were significant. In LI group, the proportion of anti-hypertensive drug use, anti-diabetic therapy, anti-platelet drug use were all higher compared with LAA group (68.2% vs. 52%, P<0.001; 33.4% vs. 23%, P<0.001; 30.6% vs. 19.7%, P<0.001). Both SBP and DBP were significantly higher in the LAA group than in the LI group (143.5 vs. 141.10, P = 0.041; 83.05 vs. 80.99, P = 0.003).Hemoglobin, total cholesterol and LDL-c were higher in the LAA group than the LI group (139.53 vs. 136.86, P = 0.002; 4.46 vs. 4.32, P = 0.042; 2.77 vs. 2.59, P = 0.001).The proportion of WML in LI group were higher than in LAA group(39.4% vs. 29.2%, P<0.001).

**Table 2:** The clinical characteristics of LI patients and LAA patients.

Variable	LAA (N = 1207)	LI (N = 566)	P value
Patients’ characteristics			
Age, year	66±13	69±12	<0.001
Male gender, n(%)	824(68.3%)	375(66.3%)	0.402
Risk factors			
Diabetes, n(%)	283(23.4%)	191(33.7%)	<0.001
Smoking, n(%)	566(47.3%)	251(44.5%)	0.291
Hypertension, n(%)	644(53.3%)	395(69.8%)	<0.001
History of TIA/CVD, n(%)	232(21.9%)	134(24%)	0.384
History of CHD, n(%)	261(21.8%)	150(26.5%)	0.033
Alcohol abuse, n(%)	458(38.4%)	196(34.8%)	0.159
Anti-hypertensive drugs, n(%)	628(52%)	386(68.2%)	<0.001
Anti-diabetic therapy, n(%)	278(23%)	189(33.4%)	<0.001
Statin use, n(%)	274(22.7%)	144(25.4%)	0.236
Anti-platelet drugs, n(%)	238(19.7%)	173(30.6%)	<0.001
Laboratory and imaging tests on admission			
Systolic blood pressure mmHg	143.5±21.33	141.10±19.70	0.041
Diastolic blood pressure mmHg	83.05±12.96	80.99±12.30	0.003
Hemoglobin, g/L	139.53±19.59	136.86±18.39	0.002
Serum albumin, g/L	39.37±4.92	39.70±5.14	0.408
Serum creatinine, μmol/L	98.90±52.59	98.75±47.44	0.695
Triglyceride, mmol/L	1.59±0.98	1.61±1.22	0.390
Total cholesterol, mmol/L	4.46±1.12	4.32±1.03	0.042
HDL-c, mmol/L	1.04±0.36	1.06±0.30	0.232
LDL-c, mmol/L	2.77±0.94	2.59±0.83	0.001
Homocysteine, μmol/L	17.93±42.78	17.16±10.69	0.064
White matter leukoaraiosis, n(%)	353(29.2%)	223(39.4%)	<0.001

Values are reported as mean±SD or n (%) of subjects. Percentages based on nonmissing values: 2 missing value of sex; 8 (1) missing value of hypertension; 10 (1) missing value of diabetes; 11 (1) missing value of history of CHD; 149 (7) missing value of history of TIA/CVD; 11 (2) missing value of smoking; 15 (3) missing value of alcohol use; 221 (37) missing value of SBP and DBP, respectively; 178 (10) missing value of hemoglobin; 172 (12) missing value of serum albumin; 14 (5) missing value of serum creatinine; 178,179,180 and 183 (14, 13, 13 and 13) missing value of triglyceride, total cholesterol, HDL-c and LDL-c, respectively; and 400 (235) missing value of homocysteine; number in LAA (number in LI stroke)

LI: lacunar infarct; LAA: large artery atherosclerosis; CHD: coronary heart disease; CVD: cerebrovascular disease; TIA; transient ischemic attack; SBP: systolic blood pressure;DBP: diastolic blood pressure; LDL: low-density lipoprotein; HDL: high-density lipoprotein.

Table 3 shows the logistic regression analysis results. In the univariate logistic analysis, history of CHD (OR = 1.295, P = 0.029), history of hypertension(OR = 2.022, P<0.001), history of diabetes(OR = 1.664, P<0.001),age (OR = 1.019, P<0.001), anti-hypertension drugs (OR = 1.981, P<0.001), anti-diabetic therapy (OR = 1.677, P<0.001), anti-platelet drugs (OR = 1.794, P<0.001), and WML (OR = 1.575, P<0.001) were found to be more strongly correlated with LI than LAA. Total cholesterol(OR = 0.884,P = 0.012), SBP(OR = 0.995,P = 0.038), DBP(OR = 0.987,P = 0.003), hemoglobin(OR = 0.993,P = 0.009) and LDL-c(OR = 0.790,P<0.001) were found to be more strongly correlated with LAA than LI. In multivariate logistic analysis, only LDL-c (OR = 0.774, P = 0.002) remained strongly associated with LAA. Hypertension (OR = 1.832, P = 0.012), and WML (OR = 1.865, P<0.001) were more strongly correlated with LI than LAA. Total cholesterol show a nonsignificant association with LAA (OR = 0.922, P = 0.130).

**Table 3:** Results of univariate and multivariate logistic analysis: clinical characteristics associated with different stroke subtypes (LI patients vs. LAA patients).

Variable	LI vs. LAA (N = 1773)			
	Unadjusted		Adjusted	
	OR (95%CI)	P	OR (95%CI)	P
Male gender	0.908(0.735, 1.124)	0.372	…	…
Hypertension	2.022(1.638, 2.504)	<0.001	1.832(1.149, 2.977)	0.012
Diabetes	1.664(1.336, 2.072)	<0.001	1.875(0.914, 4.064)	0.096
History of CHD	1.295(1.026, 1.631)	0.029	0.803(0.585, 1.096)	0.170
History of TIA/CVD	1.123(0.879, 1.429)	0.351	…	…
Anti-hypertension drugs	1.981(1.608, 2.446)	<0.001	0.861(0.513, 1.431)	0.568
Anti-diabetic therapy	1.677(1.345, 2.089)	<0.001	0.789(0.369, 1.594)	0.523
Statin use	1.158(0.917,1.460)	0.213	…	…
Anti-platelet drugs	1.794(1.427,2.253)	<0.001	1.197(0.887, 1.610)	0.238
Smoking	0.893(0.730, 1.091)	0.268	…	…
Alcohol abuse	0.856(0.694, 1.054)	0.145	0.800(0.593, 1.076)	0.141
Age	1.019(1.011, 1.027)	<0.001	1.004(0.991, 1.017)	0.538
Systolic blood pressure	0.995(0.989, 1.000)	0.038	0.993(0.984, 1.001)	0.091
Diastolic blood pressure	0.987(0.979, 0.996)	0.003	0.996(0.982, 1.011)	0.621
Hemoglobin	0.993(0.988, 0.998)	0.009	1.002(0.994, 1.009)	0.691
Serum albumin	1.013(0.992, 1.034)	0.219	…	…
Serum creatinine	1.001(0.999, 1.004)	0.343	…	…
Triglyceride	1.021(0.926, 1.123)	0.674	…	…
Total cholesterol	0.884(0.802, 0.973)	0.012	0.922(0.829, 1.023) *	0.130*
HDL-c	1.160(0.858, 1.593)	0.334	…	…
LDL-c	0.790(0.701, 0.889)	<0.001	0.774(0.658, 0.908)	0.002
Homocysteine	0.999(0.993, 1.003)	0.752	…	…
WML	1.575(1.277, 1.941)	<0.001	1.865(1.390, 2.505)	<0.001

Adjusted odds ratio (OR) models were adjusted for Hypertension, Diabetes, History of CHD, Anti-hypertension drugs, Anti-diabetic therapy, Anti-platelet drugs, Alcohol abuse, Age, SBP, DBP, Hemoglobin, LDL-c, WML in multiple logistic analyses

* Adjusted odds ratio (OR) models were adjusted for Hypertension, Diabetes, History of CHD, Anti-hypertension drugs, Anti-diabetic therapy, Anti-platelet drugs, Alcohol abuse, Age, SBP, DBP, Hemoglobin, WML in multivariate logistic analyses.

## Discussion

The present study analyzed the clinical data of patients with acute stroke treated at a tertiary hospital in Beijing and yielded the following results. First, LAA is still the main stroke subtype of all strokes, followed by LI (28.6%), and the proportions observed were similar to a previous study [17]. Second, hypertension, and WML were found to be more strongly associated with LI than with LAA. Serum LDL-c was more related to LAA than to LI.

It has been reported that the proportion of strokes due to LAA ranged from 12% to 54%, SVD 20% to 42%, cardioembolism 10% to 26%, and the combination of other specific determined and undetermined etiology subtypes 4% to 34%[17]. Different distributions of ischemic stroke subtypes among Chinese were reported[18–21]. The probable reason is the different observed population. In this study which is a hospital-based one and has been done in the capital city of China, the dominant proportion of stroke subtype is still LAA. Although there are studies show that Chinese have a much more proportion of SVD than westerners [22]. This shift of subtypes occurred during a time of rapid economic development and increasing adoption of western lifestyles in China, increased dietary fat and cholesterol intake, and possibly better hypertension control[23].

Age was thought to be a significant risk factor in both LI and LAA groups in multivariate analysis by with previous reports [24, 25]. One study found a slightly higher relation between age and small vessel disease than large vessel disease[16]. One study found that age was more related to ischemic leukoaraiosis than isolated lacunar infarction[26]. Here we found a stronger relation between age and LI than LAA in univariate analysis but not in multivariate analysis.

Hypertension is a well recognized risk factor for lacunar stroke. In this study, a stronger correlation was found between hypertension and LI compared with LAA. The result indicated that hypertension play an important role in the development of small vessel disease. One meta analysis concluded 25 studies and found that hypertension was more common among patients with LI[9]. So, it makes sense to emphasize blood pressure control in the prevention of LI. But there are also studies pointed out that stroke subtype classification bias play a role in the results for hypertension[27]. Once stroke was classified by a risk factor-free method, the stronger relationship between hypertension and lacunar versus nonlacunar infarction patients disappeared.

The relationship between lipid abnormalities and ischemic stroke is not clear, although studies focused on this point are enormous in the past[28]. Case-control studies found negative results in identifying total cholesterol as a risk factor of stroke [29, 30]. One study found a strong relationship between stroke and total cholesterol[31]. Considering stroke subtypes, the association of LAA and cholesterol is definite [28, 32, 33] Lacunar stroke has been less consistently associated with increased total cholesterol levels[33–35]. However, most studies compare LAA or LI with healthy controls. Few studies compare the difference between LAA and LI. The results we got didn’t show a possible stronger association between total cholesterol and LAA than LI either. This indicated that microatheromatous vascular disease, either in the parent vessel or in the proximal portion of the penetrating artery, played an important role in larger and symptomatic lacunar strokes[36, 37]. Prospective studies in populations are needed to clarify whether cholesterol levels are more associated with LAA than LI.

In the current study, a strong correlation between LDL-c level and LAA rather than LI was found, which is consistent with the most previous study. LDL-c level was proved to be associated with extracranial stenosis [38]. A long-term, prospective Japanese study found a significant relationship between serum LDL cholesterol level and the risk for atherothrombotic infarction, while no correlation was found between other stroke subtypes (LI and CE) and LDL-c [39]. The ARIC Study revealed that LDL-c level was asscociated with larger lacunes rather than smaller ones [40]. Because of the multiple mechanisms lacunar infarcts caused by, such as lipohyalinosis, microatheroma, arteriosclerosis, and cardioembolic occlusion. The heterogeneous roles for LDL cholesterol in the multiple pathogenesis of LI occurrence might account for the weak association between LDL cholesterol and the risk of LI. Furthermore, our findings support the idea that the pathogenesis of these two stroke subtypes is not the same.

The limitations of this study need to be considered. Patients with severe renal disease were not included in the study. Those patients have a disturbed biochemical state and multi-organ complications, such as accelerated calcific atherosclerosis, platelet dysfunction, and anemia[41]. This inclusion criteria may cause certain selection bias to our study. Other limitations were particularly those inherent to retrospective studies. Another noteworthy limitation is the lack of a community-based design, meaning that the selection process was affected by the hospital admission selection bias. Thus, further studies should be designed to be community-based, which will allow the inclusion of all stroke patients in a local area, without being subject to selection bias.

## Conclusion

In the current study, LAA is still the dominant stroke subtype in China. Consistent with previous studies, hypertension, and WML were found to be more strongly correlated with LI than LAA.LDL-c were more associated with LAA than LI.

## Supporting Information

S1 Table Correlation analysis of the variables in multivariate logistic analysis. (DOCX)
