# Supplementary material for: Comparison of Risk Factor between Lacunar Stroke and Large Artery Atherosclerosis Stroke: A Cross-Sectional Study in China
Source: PLoS One. 2016 Mar 2;11(3):e0149605. doi: 10.1371/journal.pone.0149605 (PMC4774914; doi:10.1371/journal.pone.0149605)
Supplement: S1 Table — (DOCX) [file pone.0149605.s001.docx]

| Table S1. Correlation analysis of the variables in multivariate logistic analysis | | | | | | | | | | | | | | |
| --- | --- | --- | --- | --- | --- | --- | --- | --- | --- | --- | --- | --- | --- | --- |
| r value | Hypertension | Diabetes | History of CHD | Alcohol abuse | Anti-hypertension drugs | Anti-diabetic therapy | Anti-platelet drugs | Age | SBP | DBP | Hemoglobin | Total cholesterol | LDL-c | WML |
| Hypertension | 1.000 | 0.182 | 0.115 | -0.038 | 0.708 | 0.102 | 0.204 | 0.057 | 0.171 | 0.106 | -0.022 | -0.027 | -0.061 | 0.033 |
| Diabetes | 0.182 | 1.000 | 0.074 | -0.012 | 0.088 | 0.750 | 0.109 | 0.030 | 0.038 | -0.032 | -0.090 | -0.012 | -0.005 | -0.046 |
| History of CHD | 0.115 | 0.074 | 1.000 | -0.093 | 0.129 | 0.084 | 0.061 | 0.275 | -0.086 | -0.135 | -0.139 | -0.129 | -0.127 | 0.088 |
| Alcohol abuse | -0.038 | -0.012 | -0.093 | 1.000 | -0.029 | -0.035 | -0.012 | -0.309 | 0.014 | 0.147 | 0.325 | 0.001 | 0.038 | -0.077 |
| Anti-hypertension drugs | 0.708 | 0.088 | 0.129 | -0.029 | 1.000 | 0.126 | 0.144 | 0.103 | 0.255 | 0.167 | -0.032 | -0.022 | -0.047 | 0.111 |
| Anti-diabetic therapy | 0.102 | 0.750 | 0.084 | -0.035 | 0.126 | 1.000 | 0.065 | 0.029 | 0.044 | -0.016 | -0.082 | -0.015 | -0.006 | -0.038 |
| Anti-platelet drugs | 0.204 | 0.109 | 0.061 | -0.012 | 0.144 | 0.065 | 1.000 | 0.161 | -0.001 | -0.043 | -0.101 | -0.107 | -0.098 | 0.076 |
| Age | 0.057 | 0.030 | 0.275 | -0.309 | 0.103 | 0.029 | 0.161 | 1.000 | 0.038 | -0.263 | -0.389 | -0.097 | -0.098 | 0.324 |
| SBP | 0.171 | 0.038 | -0.086 | 0.014 | 0.255 | 0.044 | -0.001 | 0.038 | 1.000 | 0.591 | 0.087 | 0.149 | 0.137 | 0.095 |
| DBP | 0.106 | -0.032 | -0.135 | 0.147 | 0.167 | -0.016 | -0.043 | -0.263 | 0.591 | 1.000 | 0.299 | 0.150 | 0.148 | -0.013 |
| Hemoglobin | -0.022 | -0.090 | -0.139 | 0.325 | -0.032 | -0.082 | -0.101 | -0.389 | 0.087 | 0.299 | 1.000 | 0.166 | 0.185 | -0.114 |
| Total cholesterol | -0.027 | -0.012 | -0.129 | 0.001 | -0.022 | -0.015 | -0.107 | -0.097 | 0.149 | 0.150 | 0.166 | 1.000 | 0.901 | 0.024 |
| LDL-c | -0.061 | -0.005 | -0.127 | 0.038 | -0.047 | -0.006 | -0.098 | -0.098 | 0.137 | 0.148 | 0.185 | 0.901 | 1.000 | 0.036 |
| WML | 0.033 | -0.046 | 0.088 | -0.077 | 0.111 | -0.038 | 0.076 | 0.324 | 0.095 | -0.013 | -0.114 | 0.024 | 0.036 | 1.000 |
|  |  |  |  |  |  |  |  |  |  |  |  |  |  |  |
|  |  |  |  |  |  |  |  |  |  |  |  |  |  |  |
|  |  |  |  |  |  |  |  |  |  |  |  |  |  |  |
| P value | Hypertension | Diabetes | History of CHD | Alcohol abuse | Anti-hypertension drugs | Anti-diabetic therapy | Anti-platelet drugs | Age | SBP | DBP | Hemoglobin | Total cholesterol | LDL-c | WML |
| Hypertension | 1.000 | 0.000 | 0.000 | 0.112 | 0.000 | 0.000 | 0.000 | 0.016 | 0.000 | 0.000 | 0.380 | 0.292 | 0.016 | 0.160 |
| Diabetes | 0.000 | 1.000 | 0.002 | 0.606 | 0.000 | 0.000 | 0.000 | 0.210 | 0.144 | 0.215 | 0.000 | 0.637 | 0.850 | 0.053 |
| History of CHD | 0.000 | 0.002 | 1.000 | 0.000 | 0.000 | 0.000 | 0.010 | 0.000 | 0.001 | 0.000 | 0.000 | 0.000 | 0.000 | 0.000 |
| Alcohol abuse | 0.112 | 0.606 | 0.000 | 1.000 | 0.220 | 0.142 | 0.628 | 0.000 | 0.595 | 0.000 | 0.000 | 0.975 | 0.130 | 0.001 |
| Anti-hypertension drugs | 0.000 | 0.000 | 0.000 | 0.220 | 1.000 | 0.000 | 0.000 | 0.000 | 0.000 | 0.000 | 0.208 | 0.386 | 0.065 | 0.000 |
| Anti-diabetic therapy | 0.000 | 0.000 | 0.000 | 0.142 | 0.000 | 1.000 | 0.007 | 0.218 | 0.088 | 0.532 | 0.001 | 0.544 | 0.818 | 0.113 |
| Anti-platelet drugs | 0.000 | 0.000 | 0.010 | 0.628 | 0.000 | 0.007 | 1.000 | 0.000 | 0.974 | 0.091 | 0.000 | 0.000 | 0.000 | 0.001 |
| Age | 0.016 | 0.210 | 0.000 | 0.000 | 0.000 | 0.218 | 0.000 | 1.000 | 0.135 | 0.000 | 0.000 | 0.000 | 0.000 | 0.000 |
| SBP | 0.000 | 0.144 | 0.001 | 0.595 | 0.000 | 0.088 | 0.974 | 0.135 | 1.000 | 0.000 | 0.001 | 0.000 | 0.000 | 0.000 |
| DBP | 0.000 | 0.215 | 0.000 | 0.000 | 0.000 | 0.532 | 0.091 | 0.000 | 0.000 | 1.000 | 0.000 | 0.000 | 0.000 | 0.624 |
| Hemoglobin | 0.380 | 0.000 | 0.000 | 0.000 | 0.208 | 0.001 | 0.000 | 0.000 | 0.001 | 0.000 | 1.000 | 0.000 | 0.000 | 0.000 |
| Total cholesterol | 0.292 | 0.637 | 0.000 | 0.975 | 0.386 | 0.544 | 0.000 | 0.000 | 0.000 | 0.000 | 0.000 | 1.000 | 0.000 | 0.332 |
| LDL-c | 0.016 | 0.850 | 0.000 | 0.130 | 0.065 | 0.818 | 0.000 | 0.000 | 0.000 | 0.000 | 0.000 | 0.000 | 1.000 | 0.151 |
| WML | 0.160 | 0.053 | 0.000 | 0.001 | 0.000 | 0.113 | 0.001 | 0.000 | 0.000 | 0.624 | 0.000 | 0.332 | 0.151 | 1.000 |
